# Supplementary material for: OpenMM 7: Rapid development of high performance algorithms for molecular dynamics
Source: PLoS Comput Biol. 2017 Jul 26;13(7):e1005659. doi: 10.1371/journal.pcbi.1005659 (PMC5549999; doi:10.1371/journal.pcbi.1005659)
Supplement: S1 Source Code — (ZIP) [file pcbi.1005659.s002.zip › OpenMM-7.0.1-Source/docs/api-c++/generated/OpenMM.MonteCarloAnisotropicBarostat.html]

MonteCarloAnisotropicBarostat — OpenMM @OPENMM\_MAJOR\_VERSION@.@OPENMM\_MINOR\_VERSION@ documentation


# MonteCarloAnisotropicBarostat¶

*class* `OpenMM::``MonteCarloAnisotropicBarostat`¶
:   This class uses a Monte Carlo algorithm to adjust the size of the periodic box, simulating the effect of constant pressure.

    This class is similar to `MonteCarloBarostat`, but each Monte Carlo move is applied to only one axis of the periodic box (unlike `MonteCarloBarostat`, which scales the entire box isotropically). This means that the box may change shape as well as size over the course of the simulation. It also allows you to specify a different pressure for each axis of the box, or to keep the box size fixed along certain axes while still allowing it to change along others.

    This class assumes the simulation is also being run at constant temperature, and requires you to specify the system temperature (since it affects the acceptance probability for Monte Carlo moves). It does not actually perform temperature regulation, however. You must use another mechanism along with it to maintain the temperature, such as `LangevinIntegrator` or `AndersenThermostat`.

    Methods

    |  |  |
    | --- | --- |
    | `MonteCarloAnisotropicBarostat` | Create a `MonteCarloAnisotropicBarostat`. |
    | `getDefaultPressure` | Get the default pressure (in bar). |
    | `setDefaultPressure` | Set the default pressure acting on the system. |
    | `getScaleX` | Get whether to allow the X dimension of the periodic box to change size. |
    | `getScaleY` | Get whether to allow the Y dimension of the periodic box to change size. |
    | `getScaleZ` | Get whether to allow the Z dimension of the periodic box to change size. |
    | `getFrequency` | Get the frequency (in time steps) at which Monte Carlo pressure changes should be attempted. |
    | `setFrequency` | Set the frequency (in time steps) at which Monte Carlo pressure changes should be attempted. |
    | `getTemperature` | Get the temperature at which the system is being maintained, measured in Kelvin. |
    | `setTemperature` | Set the temperature at which the system is being maintained. |
    | `getRandomNumberSeed` | Get the random number seed. |
    | `setRandomNumberSeed` | Set the random number seed. |
    | `usesPeriodicBoundaryConditions` | Returns whether or not this force makes use of periodic boundary conditions. |

    *const* std::string &`PressureX`()¶
    :   This is the name of the parameter which stores the current pressure acting on the X-axis (in bar).

    *const* std::string &`PressureY`()¶
    :   This is the name of the parameter which stores the current pressure acting on the Y-axis (in bar).

    *const* std::string &`PressureZ`()¶
    :   This is the name of the parameter which stores the current pressure acting on the Z-axis (in bar).

    `MonteCarloAnisotropicBarostat`(*const* Vec3 &*defaultPressure*, double *temperature*, bool *scaleX* = true, bool *scaleY* = true, bool *scaleZ* = true, int *frequency* = 25)¶
    :   Create a `MonteCarloAnisotropicBarostat`.

        |  |  |
        | --- | --- |
        | Parameters: |  |

        - **defaultPressure** – The default pressure acting on each axis (in bar)
        - **temperature** – the temperature at which the system is being maintained (in Kelvin)
        - **scaleX** – whether to allow the X dimension of the periodic box to change size
        - **scaleY** – whether to allow the Y dimension of the periodic box to change size
        - **scaleZ** – whether to allow the Z dimension of the periodic box to change size
        - **frequency** – the frequency at which Monte Carlo pressure changes should be attempted (in time steps)

    *const* Vec3 &`getDefaultPressure`() *const*¶
    :   Get the default pressure (in bar).

        |  |  |
        | --- | --- |
        | Returns: | the default pressure acting along each axis, measured in bar. |

    void `setDefaultPressure`(*const* Vec3 &*pressure*)¶
    :   Set the default pressure acting on the system. This will affect any new Contexts you create, but not ones that already exist.

        |  |  |
        | --- | --- |
        | Parameters: |  |

        - **pressure** – the default pressure acting on the system, measured in bar.

    bool `getScaleX`() *const*¶
    :   Get whether to allow the X dimension of the periodic box to change size.

    bool `getScaleY`() *const*¶
    :   Get whether to allow the Y dimension of the periodic box to change size.

    bool `getScaleZ`() *const*¶
    :   Get whether to allow the Z dimension of the periodic box to change size.

    int `getFrequency`() *const*¶
    :   Get the frequency (in time steps) at which Monte Carlo pressure changes should be attempted. If this is set to 0, the barostat is disabled.

    void `setFrequency`(int *freq*)¶
    :   Set the frequency (in time steps) at which Monte Carlo pressure changes should be attempted. If this is set to 0, the barostat is disabled.

    double `getTemperature`() *const*¶
    :   Get the temperature at which the system is being maintained, measured in Kelvin.

    void `setTemperature`(double *temp*)¶
    :   Set the temperature at which the system is being maintained.

        |  |  |
        | --- | --- |
        | Parameters: |  |

        - **temp** – the system temperature, measured in Kelvin.

    int `getRandomNumberSeed`() *const*¶
    :   Get the random number seed. See `setRandomNumberSeed()` for details.

    void `setRandomNumberSeed`(int *seed*)¶
    :   Set the random number seed. It is guaranteed that if two simulations are run with different random number seeds, the sequence of Monte Carlo steps will be different. On the other hand, no guarantees are made about the behavior of simulations that use the same seed. In particular, Platforms are permitted to use non-deterministic algorithms which produce different results on successive runs, even if those runs were initialized identically.

        If seed is set to 0 (which is the default value assigned), a unique seed is chosen when a `Context` is created from this `Force`. This is done to ensure that each `Context` receives unique random seeds without you needing to set them explicitly.

    bool `usesPeriodicBoundaryConditions`() *const*¶
    :   Returns whether or not this force makes use of periodic boundary conditions.

        |  |  |
        | --- | --- |
        | Returns: | true if force uses PBC and false otherwise |

High performance molecular simulation on GPUs


### Navigation

- Core Objects
- Forces
- Integrators
- Extras

©2015, Stanford University and the Authors.
|
Powered by Sphinx 1.3.1
& Alabaster 0.7.7
|
Page source
